# A Selective PMCA Inhibitor Does Not Prolong the Electroolfactogram in Mouse

**DOI:** 10.1371/journal.pone.0037148

**Published:** 2012-05-15

**Authors:** Edwin R. Griff, Nancy K. Kleene, Steven J. Kleene

**Affiliations:** 1 Department of Biological Sciences, University of Cincinnati, Cincinnati, Ohio, United States of America; 2 Department of Cancer and Cell Biology, University of Cincinnati, Cincinnati, Ohio, United States of America; Monell Chemical Senses Center, United States of America

## Abstract

**Background:**

Within the cilia of vertebrate olfactory receptor neurons, Ca^2+^ accumulates during odor transduction. Termination of the odor response requires removal of this Ca^2+^, and prior evidence suggests that both Na^+^/Ca^2+^ exchange and plasma membrane Ca^2+^-ATPase (PMCA) contribute to this removal.

**Principal Findings:**

In intact mouse olfactory epithelium, we measured the time course of termination of the odor-induced field potential. Replacement of mucosal Na^+^ with Li^+^, which reduces the ability of Na^+^/Ca^2+^ exchange to expel Ca^2+^, prolonged the termination as expected. However, treating the epithelium with the specific PMCA inhibitor caloxin 1b1 caused no significant increase in the time course of response termination.

**Conclusions:**

Under these experimental conditions, PMCA does not contribute detectably to the termination of the odor response.

## Introduction

In vertebrates, transduction of odorous stimuli occurs in the cilia of olfactory receptor neurons (reviewed in [1], [2]). Both initiation and termination of the receptor current depend on intraciliary Ca^2+^. During initiation, a transduction cascade causes a depolarizing influx of Ca^2+^ and Na^+^ into the cilium through cyclic-nucleotide-gated (CNG) channels. Intraciliary Ca^2+^ then gates anoctamin-2 Cl^−^ channels (reviewed in [3]), which allow an efflux of Cl^−^ that further depolarizes the neuron. Ca^2+^ also plays key roles in terminating the receptor current. Together with calmodulin, Ca^2+^ reduces the sensitivity of the CNG channels to their gating ligand, cAMP. Reductions in intraciliary Ca^2+^ also allow the Cl^−^ channels to close, which completes the termination of the receptor current.

Evidence has suggested a range of mechanisms by which Ca^2+^ might leave the cilium. Modeling suggests that simple diffusion into the dendrite is too slow to account for the observed response termination [4]. In amphibians [5]–[8], rat [9], and mouse [10]–[12], Ca^2+^ efflux is coupled to an influx of Na^+^ (Na^+^/Ca^2+^ exchange). Other studies in toad [8], mouse [10], [11], and salamander [13] suggest that Ca^2+^ is also pumped out of the cilium by a plasma membrane Ca^2+^-ATPase (PMCA). Much but not all of the evidence that indicates a role for PMCA in response recovery derives from the use of PMCA inhibitors [8], [10], [11], [13]. As noted elsewhere [14], [15], the traditional inhibitors, eosin and vanadate, inhibit not just PMCA but all ATPases, including the Na^+^, K^+^-ATPase. Na^+^, K^+^-ATPase is expressed in olfactory cilia [16], [17]. Inhibition of the Na^+^, K^+^-ATPase during the odor response may allow intraciliary Na^+^ to accumulate, which in turn could prevent Na^+^/Ca^2+^ exchange from expelling Ca^2+^ [4]. Thus an inhibitor chosen to study PMCA may in fact modify Ca^2+^ transport via an indirect effect on Na^+^/Ca^2+^ exchange. A further complication is that the PMCA inhibitor carboxyeosin modulates the ciliary CNG channels [18].

More selective inhibitors of PMCA are now available [14], [15]. The caloxins are a family of peptides engineered to bind selectively to extracellular domains of PMCA proteins. We have found that applying a caloxin to mouse olfactory epithelium has no effect that is consistent with a role for PMCA in Ca^2+^ extrusion following the odor response.

## Materials and Methods

### Animal preparation

All experiments were approved by the University of Cincinnati's Institutional Animal Care and Use Committee (protocol 04-11-05-01) and conducted in accordance with the recommendations in the “Guide for the Care and Use of Laboratory Animals” of the National Institutes of Health.

### Animal preparation

All efforts were made to minimize animal suffering and to reduce the number of animals used. Experiments were performed on adult mice from the inbred FVB/N genetic background. Animals were euthanized with CO_2_ and decapitated, and the lower jaw was removed. After trimming skin and muscle, the caudal 25% of the brain was removed with scissors, the dorsal aspect of the nasal septum cut with spring scissors, the nasal bone removed, and the skull divided along the midline with a scalpel such that the nasal septum remained with one half of the skull. The septum was carefully removed and the exposed olfactory turbinates surrounded by gauze threads so that solutions applied were retained covering the turbinates. The half head was glued into an organ culture dish with water filling the trough surrounding but not contacting the tissue and placed in a Faraday cage for recording. The other side of the head was kept on ice for subsequent use. A silver-silver chloride reference electrode inserted into the posterior aspect of the remaining brain was connected to ground. The dissection and recording area was continuously humidified.

### Solutions

The Ringer solution contained (in mM): 140 NaCl, 5 KCl, 2 CaCl_2_, 2 MgCl_2_, 2 Na pyruvate, 5 HEPES, 9.4 D-glucose, adjusted to pH 7.4 with NaOH. For the Li^+^-replaced Ringer, LiCl was substituted for NaCl and the pH was adjusted with LiOH. Caloxin 1b1 (amino-acid sequence TAWSEVLHLLSRGGG-amide [14], [19]) was synthesized by Alpha Diagnostic International Inc. (San Antonio, TX, USA). A 10 mM stock solution made in 25% ethanol was diluted to 100 µM or 500 µM in Ringer. In previous studies, the K_i_ values for inhibition of PMCA by caloxin 1b1 were 105 µM (PMCA1), 167 µM (PMCA2), 274 µM (PMCA3), and 45 µM (PMCA4) [19], [20]. These studies indicate that 100 µM caloxin 1b1 should inhibit PMCA by 27 to 68%, depending on the isoform; 500 µM caloxin should inhibit by 64 to 92%.

### Recording Protocol

**Figure 1:**
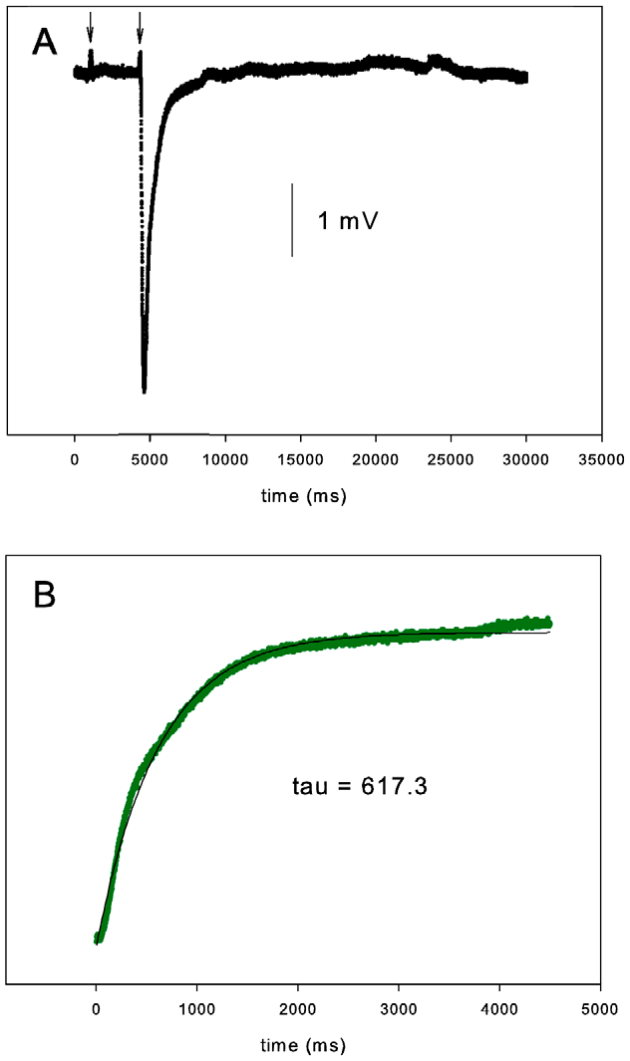
Control EOG. (A) At the first arrow, a 100-ms stimulus of unodorized air was applied to the turbinate. At the second arrow, a 100-ms stimulus of isoamyl acetate was applied. The amplitude was 4.4 mV. (B) The first 4500 ms of the recovery from the minimum of the EOG in A are plotted (green) along with a regression curve (black; y = A*e* ^−t/τ^+c). The R^2^ for the fit was 0.993 and the time constant of the recovery, τ, was 617 ms.

We measured the electroolfactogram (EOG), an odor-induced field potential at the surface of the olfactory epithelium [21], [22]. The EOG was recorded between a glass microelectrode filled with Ringer that touched the surface of a turbinate and the reference electrode. The micromanipulator (Narishige International, East Meadow, NY, USA) allowed the microelectrode to be withdrawn and repositioned repeatedly to the same location on a turbinate. Electrical signals were amplified by a high-impedance preamplifier (AK-47LN, Metametrics, Cambridge, MA, USA). The amplified signal was digitized and visualized on a PC using Igor Pro 4 software (Wavemetrics, Portland, OR, USA). 100-ms pulses of air at 10 psi, odorized by passing through a vial containing 99% isoamyl acetate, were directed at the turbinates using a Picospritzer II (Parker Instrumentation, Fairfield, NJ, USA). A 100-ms pulse of non-odorized air was applied about 5 s before the odorant. Since the response evoked by non-odorized air was a small fraction of the response to the odorant (Fig. 1A), the EOG amplitudes were not corrected for this. Our criteria for including the EOGs recorded were that the control EOG was at least 2 mV in amplitude and had a time constant of recovery from the peak (minimum) of less than 1 s (see below). Successive stimuli were separated by at least 2 min.

**Figure 2:**
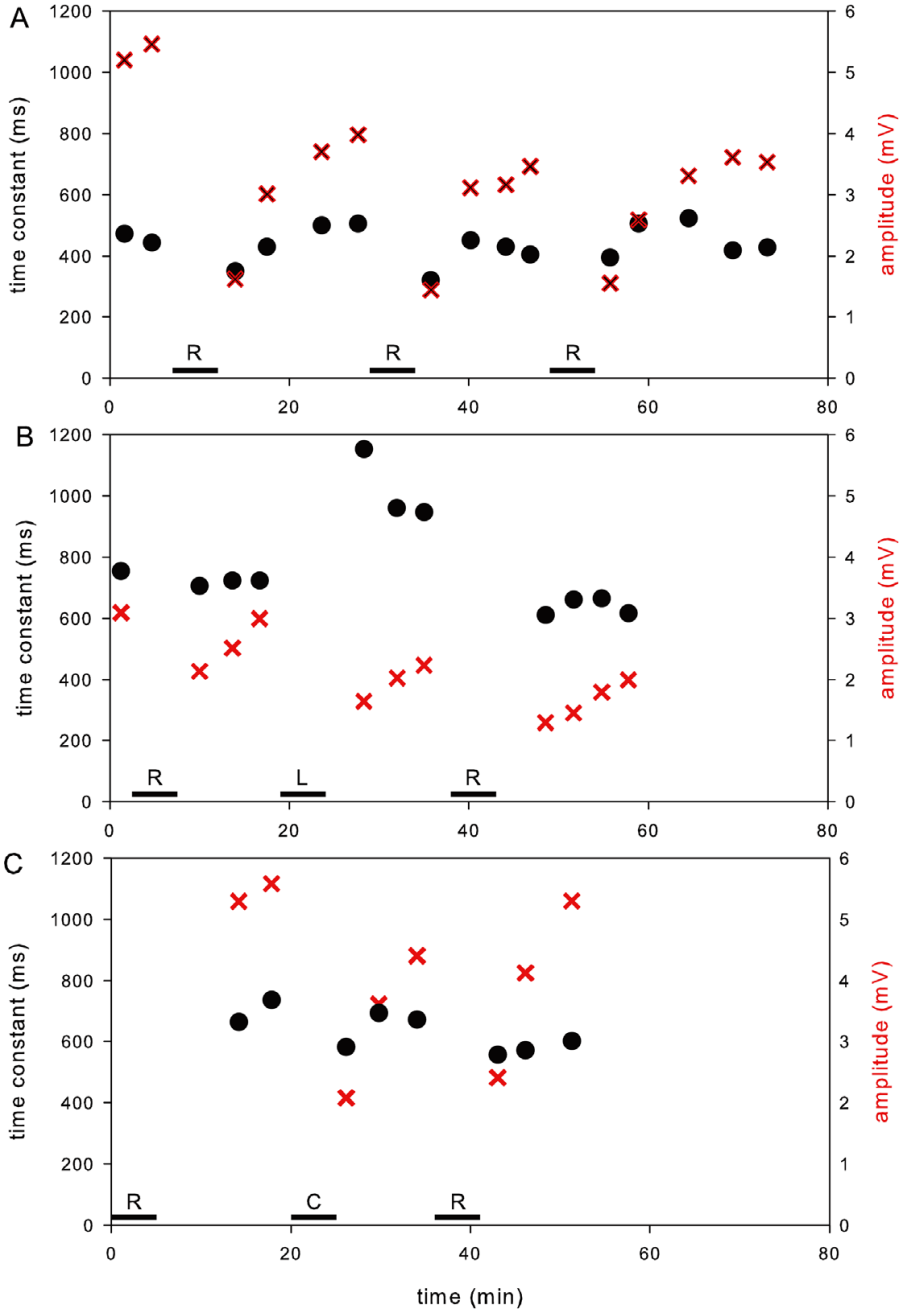
EOG changes over time. The EOG was recorded before and after each of several treatments. Each treatment consisted of applying solution to the tissue for about 5 min (horizontal bars). The amplitudes of the EOG (X's) and the time constants (filled circles) were measured and plotted. (A) 3 repeated treatments with control Ringer (R). The EOG amplitudes transiently decreased after each treatment while the time constant changed little. (B) The EOG was recorded before and after a Ringer treatment (R), then treatment with a Li^+^-replaced Ringer (L), and then after rinsing the tissue with Ringer and a Ringer treatment (R). The Li^+^-replaced Ringer caused an increase in the time constant. (C) The EOG was recorded after a Ringer treatment (R), then treatment with Ringer containing 100 µM caloxin (C), and then after rinsing the tissue with Ringer and a subsequent Ringer treatment (R). The time constant changed little after caloxin treatment.

After locating an area of a turbinate that produced an acceptable EOG, the recording electrode was withdrawn. Ringer was applied to the turbinates, left for about 5 min covering the turbinates, and then wicked away. The electrode was repositioned in contact with the same area and several control EOGs were recorded. After applying and wicking a solution from a turbinate, the amplitude of the EOG was initially reduced but recovered substantially (see Fig. 2). Wicking was not able to remove all the added liquid and the change in amplitude was presumably due to the increased thickness of the unstirred layer covering the receptive surface. This layer of liquid could reduce access of the odorant to the tissue and/or could reduce the density of EOG currents. As liquid evaporated or drained off, the EOG amplitude recovered. The amplitude and time constant of the third EOG following a Ringer treatment were usually used for analysis (e.g. Table 1).

**Table 1:** Effects of Li^+^ and caloxin on termination of the odor response.

	control		treatment		control	
	amp (mV)	τ (ms)	amp (mV)	τ (ms)	amp (mV)	τ (ms)
Lithium						
	5.8	796	2.8	1378	2.7	858
	4.4	617	2.2	1000	2.7	745
	5.1	556	1.1	926	2.0	817
	3.8	513	1.1	788	1.3	797
	5.7	593	3.6	791	3.5	668
*	3.0	723	2.2	947	2.0	615
caloxin						
	4.8	798	5.3	788		
	2.7	745	2.0	659	2.8	644
	2.5	910	3.3	598	3.9	574
	3.1	809	3.2	611	3.8	536
	4.6	488	4.4	406		
	5.0	598	5.4	737	3.5	828
	2.9	936	2.4	843	2.2	675
	4.4	776	5.9	820	8.0	814
*	5.6	735	4.4	672	5.3	601

For each experiment we show the amplitude (amp) in mV and the time constant τ of the recovery from the EOG minimum in ms. The data in the rows marked with an * are included in Figure 2.

The electrode was withdrawn again, and the test solution, either Li^+^-replaced Ringer or Ringer containing 100 µM or 500 µM caloxin, was applied and left for about 5 min covering the turbinates and then wicked. The electrode was repositioned again in contact with the same area and EOGs recorded as above. The recording electrode was withdrawn again and the turbinates were rinsed 3 times with Ringer and then additional Ringer applied for 5 min, wicked, and the EOG recorded again. For a given test solution, only one such experimental series was done on a given turbinate. In some experiments, the second side of the head was also used. The control EOG in every run met the criteria given above for amplitude and time constant.

Regardless of the treatment, the recovery of the EOG amplitude from its peak after application of a solution was somewhat variable. One likely source of variability was the exact position of the recording electrode on a turbinate. For example, when recording from a more caudal position on a turbinate, it may have been more difficult to get rid of excess fluid. This might have led to a decrease in the amplitude of the EOG. Based on visual inspection, the rostral portions of turbinates tended to dry out sooner than the caudal portions.

### Analysis

The time constant τ of the recovery from the peak (minimum) of the EOG was calculated by fitting an exponential function y = A*e* ^−t/τ^+c to the 4.5 s of recording following the peak (minimum) of the EOG using SigmaPlot (Systat Software, Chicago, IL, USA). Amplitudes and time constants measured in the control (Ringer) condition and the experimental condition were compared using a paired *t*-test; p values less than 0.05 were considered significant. Results of repeated experiments are reported as mean ± SD.

## Results

EOGs were recorded from 17 different turbinates from 5 mice, with both sides of the head used in 2 of the mice. A typical control EOG with an amplitude of 4.4 mV is shown in Fig. 1A. Fig. 1B shows the 4.5 s of recovery following the minimum and the fit of the equation y = A*e* ^−t/τ^+c to that recovery. The time constant τ of the recovery was 617 ms. For all experimental runs (*n* = 20), the control amplitudes ranged from 2.5 to 8.0 mV with a mean of 4.5±1.3 mV, and τ ranged from 397 to 910 ms with a mean of 637±171 ms. Control EOGs meeting the criteria described above often could be recorded for at least 5 hr after the mouse was euthanized. These EOG amplitudes were not maximal, since increasing the pressure on the Picospritzer and therefore the amount of odorant delivered to the tissue could more than double the amplitude.

Figure 2A shows changes in the EOG amplitude and time constant τ over time with 3 sequential treatments with Ringer (horizontal bars labeled R). In each treatment, Ringer remained on the tissue for about 5 min and was then removed by wicking. As in this example, the amplitudes (shown as X's in the figure) typically decreased after each treatment and recovered. In some cases the response recovered completely and in other cases partially. By the third response after wicking away the solution covering the tissue, the amplitude approached its maximum recovery. On the other hand τ (filled circles) did not change much following Ringer treatments.

Figure 2B shows changes in the EOG amplitude and τ after treating first with Ringer, then with Li^+^-replaced Ringer, and then after rinsing again with normal Ringer. After Ringer treatment, τ did not change much (as in Fig. 2A). However, after Li^+^ treatment, τ noticeably increased and then recovered after rinsing the tissue with Ringer containing the normal Na^+^ concentration. As in Fig. 2A, the amplitude decreased after either solution and partially recovered by the third response after wicking away the solution.

Figure 2C shows changes in the EOG amplitude and τ after treating first with Ringer, then with Ringer containing 100 µM caloxin 1b1, and then after rinsing the tissue with Ringer and a subsequent Ringer treatment. As in Fig. 2A and 2B above, after each treatment, the amplitude of the EOG decreased and at least partially recovered. As in Fig. 2A, τ did not change with Ringer treatment. It also was little changed by caloxin treatment.

Table 1 shows the data for 6 experiments with Li^+^ and 9 experiments with caloxin. Treatment with Li^+^ decreased the mean amplitude from 4.6±1.0 mV to 2.2±0.9 mV and increased the mean time constant τ from 633±107 ms to 972±216 ms; both changes were statistically significant (two-tailed paired *t* test, p = 0.002). On average, τ increased to 153% of control (range 131% to 173%). Treatment with 100 µM caloxin did not significantly change the amplitude or τ relative to control. The mean amplitude in caloxin was 4.0±1.4 mV compared to 4.0±1.2 mV in control. The mean τ in caloxin was 682±136 ms compared to 755±141 ms in Ringer. In caloxin, τ decreased to 90.3% of control (range 65.7% to 123.2%), but the change was not significant (two-tailed paired *t* test, p = 0.13). We also tested a higher concentration of caloxin (500 µM, *n* = 5). This decreased τ to 371±17 ms compared to 431±24 ms in Ringer. The decrease was significant (two-tailed paired *t* test, p = 0.013).

## Discussion

To investigate whether PMCA contributes to termination of the odor response, we tested one of the caloxins, a class of selective inhibitors of PMCA [14], [15]. Of the several caloxins available, we selected caloxin 1b1, which has activity against all four PMCA isoforms [20]. In intact mouse olfactory epithelium, the recovery time of the response to the odorant isoamyl acetate was measured. The recovery time was taken to reflect the time needed to expel Ca^2+^ from the olfactory cilia; this relation has been directly established in isolated olfactory receptor neurons [13]. When Na^+^ in the solution bathing the epithelium was replaced with Li^+^, the recovery time increased significantly. This is explained by a reduction in Na^+^/Ca^2+^ exchange activity and is consistent with previous results in isolated ORNs [5]–[9], [11], [12]. If Ca^2+^ clearance by PMCA had contributed to termination of the odor response, then inhibition of PMCA would have increased the recovery time. However, application of the inhibitor caloxin caused no significant increase in the recovery time. In fact, a high dose of caloxin (500 µM) caused a small but significant decrease in the recovery time. It thus seems unlikely that PMCA plays a major role in termination of the odor response in intact mouse olfactory epithelium.

Substantial prior evidence indicates that PMCA may facilitate the expulsion of ciliary Ca^2+^ during termination of the odor response. All four principal isoforms of PMCA are expressed in mouse ORNs, and some isoforms are found in the cilia [23]. ATP-dependent Ca^2+^ transport has been demonstrated in membrane vesicles from a preparation enriched in olfactory cilia [8]. Recovery from the response to IBMX, which simulates odor stimuli, is slowed in ORNs of mice lacking PMCA2 [11].

Additional evidence has come from pharmacological studies using carboxyeosin. In isolated ORNs [8], [11], [13] and intact olfactory epithelium [10], carboxyeosin (10 to 90 µM) slows recovery from stimuli that simulate the odor response. Since carboxyeosin inhibits PMCA (IC_50_ = 20 nM [24]), it should cause intraciliary Ca^2+^ that accumulates during the response to be more slowly expelled. The retained Ca^2+^ would then continue to gate the Cl^−^ channels, accounting for the prolonged receptor current. However, one must consider whether a prolonged response might in part reflect effects of carboxyeosin on other transduction proteins. Concentrations of carboxyeosin as low as 0.1 µM significantly increase current through the ciliary CNG transduction channels near the resting potential [18]. Since this effect is slow to reverse, it could prolong the response recovery. Inhibition of Na^+^/Ca^2+^ exchange by carboxyeosin would likely do the same. Saidu et al. [11] described a protocol designed to prevent such inhibition. As noted by those authors, eosin does not inhibit Na^+^/Ca^2+^ exchange at concentrations up to 20 µM [25]. Since carboxyeosin is closely related to eosin, it is also likely to be specific for PMCA at sufficiently low concentration.

In theory, an indirect effect on Na^+^/Ca^2+^ exchange could also explain the effect of carboxyeosin [18]. Eosin inhibits all ATPases [14], [15], including the Na^+^, K^+^-ATPase, and it is likely that the closely related compound carboxyeosin does as well. The EC_50_ for inhibition of the Na^+^, K^+^-ATPase by eosin is 19 µM [26]. During response recovery, Na^+^/Ca^2+^ exchange allows the cilium to expel Ca^2+^ and take up Na^+^. Unless Na^+^, K^+^-ATPase can concurrently expel Na^+^ from the cilium, it is predicted that Na^+^ will accumulate, preventing further expulsion of Ca^2+^ via Na^+^/Ca^2+^ exchange [4]. In other words, carboxyeosin might prolong the response by inhibiting Na^+^, K^+^-ATPase (and indirectly Na^+^/Ca^2+^ exchange) rather than by inhibiting PMCA. Other pharmacological designs must also consider this ambiguity. Vanadate [13] prolongs the recovery but, like eosin, inhibits all ATPases [14], [15]. Reducing cytoplasmic ATP prolongs the odor response [8], but again this could be from an effect on either PMCA or Na^+^, K^+^-ATPase. The ambiguities in these pharmacological approaches were our motive for testing the selective PMCA inhibitor caloxin.

There is now evidence both to favor and oppose a significant role for PMCA in termination of the odor response. Evidence in favor includes the clear slowing of the odor response recovery in the neurons of mice lacking PMCA2 [11]. In the same study, the response recovery in wild-type mice was also slowed by 10 µM carboxyeosin, which is not expected to inhibit Na^+^/Ca^2+^ exchange [11]. Other supportive evidence comes from a study of response recovery in neurons of the fire salamander [13]. This experimental protocol was specifically designed to account for all of the likely non-specific effects of carboxyeosin. Replacement of extracellular Na^+^ prolonged the response recovery, and the extent of this change was taken to reflect the maximum possible contribution of Na^+^/Ca^2+^ exchange. Carboxyeosin caused an even greater prolongation, and the additional slowing of the recovery by carboxyeosin could not have arisen from blocking Na^+^/Ca^2+^ exchange (directly or indirectly via Na^+^, K^+^-ATPase). Carboxyeosin was applied at a point when the CNG channels are closed, so a non-specific effect on the channels was also excluded. It could therefore be concluded that the effect of carboxyeosin in part reflected an inhibition of PMCA. Two studies have now failed to detect an involvement of PMCA in response recovery. In frog olfactory cilia, no ATP-dependent Ca^2+^ transport was detectable as Ca^2+^ entered olfactory cilia [18]. In the present study, a specific PMCA inhibitor did not prolong the odor response (Fig. 2C, Table 1).

When comparing these various studies, it is important to consider the different preparations used. In particular, there are probably significant functional differences between isolated neurons and intact epithelium. This report is the first to assess the effects of chemical modulators of PMCA or Na^+^/Ca^2+^ exchange on the olfactory field potential (EOG) in intact epithelium. As expected, replacing mucosal Na^+^ with Li^+^ prolonged the recovery phase of the odor response. However, the effect of Na^+^ replacement in isolated ORNs is usually greater than the effect we observed in epithelium (refs. [6]–[8], [11], [12]; but see also [13]). Three explanations for this difference seem likely. First, the mucus secreted by the epithelium may partially counter the replacement of other cations by Li^+^ applied to the epithelial surface. Second, epithelial perfusion should primarily affect the membranes exposed to the mucus, whereas the entire cytoplasmic membrane of an isolated ORN is usually exposed to the bath solution provided. (When suction pipette recording is applied to isolated neurons, only the sensory endings are exposed [13].) Finally, recent evidence suggests that the Cl^−^ current itself may depend greatly on experimental conditions [27]. Deletion of the Cl^−^ channel anoctamin-2 caused no reduction in the EOG when the odor stimulus was provided as a vapor. When the epithelium was perfused with a Na^+^-containing Ringer, the amplitude of the EOG was reduced by ∼40% after deleting anoctamin-2. In isolated ORNs, deletion of anoctamin-2 reduced a simulated odor response by ∼90%. It has therefore been suggested that Cl^−^ currents during the odor response may be large only in extracellular solutions with concentrations of monovalent ions that are higher than those in the mucus [27]. Such solutions would promote ciliary accumulation of Cl^−^ via NKCC1 transport. In the native tissue, it has never been clear whether the ionic concentrations measured at the neuronal ending could support Cl^−^ uptake via NKCC1 or Ca^2+^ extrusion via Na^+^/Ca^2+^ exchange [1]. The test we presently describe for PMCA activity may be less sensitive simply because Ca^2+^ accumulation and clearance are less prominent in intact epithelium. Most physiological evidence of a significant role for PMCA in the odor response has been obtained in isolated neurons [8], [11], [13].

Another difficulty in comparing the existing PMCA studies is the diversity of species chosen. As discussed by Antolin et al. [13], the activity of PMCA in terminating the odor response may differ between terrestrial and aquatic species. Finally, one can evaluate whether a given study was able to discriminate between PMCA in the cilium and PMCA in the soma. PMCA is present in both compartments [8], [11], [23]. Two physiological studies have focused on the cilia. In suction pipette recordings of isolated salamander neurons, reagents applied to the sensory endings indicated PMCA activity during recovery from a simulated odor response [13]. In isolated frog olfactory cilia, though, no ATP-dependent effect on Ca^2+^ influx was detectable [18]. As noted elsewhere [11], ciliary PMCA probably contributes to maintaining a low resting concentration of cytoplasmic Ca^2+^. To what extent it facilitates rapid termination of the odor response now appears to depend significantly on the particular species and preparation examined.
